# Second-Generation Polyamidoamine Dendrimer Conjugated with Oligopeptides Can Enhance Plasmid DNA Delivery In Vitro

**DOI:** 10.3390/molecules28227644

**Published:** 2023-11-17

**Authors:** Seongyeon Kim, Le Thi Thuy, Jeil Lee, Joon Sig Choi

**Affiliations:** Department of Biochemistry, Chungnam National University, 99 Daehak-ro, Yuseong-gu, Daejeon 34134, Republic of Korea; sykim96500@gmail.com (S.K.); ltthuy1990@gmail.com (L.T.T.); jeillee@hongik.ac.kr (J.L.)

**Keywords:** PAMAM, gene carrier, RRHRH, oligopeptide, transfection

## Abstract

Poly(amidoamine) (PAMAM) dendrimers have attracted considerable attention in the field of gene therapy due to their flexibility in introducing different functional moieties and reduced toxicity at low generations. However, their transfection efficiency remains a limitation. Therefore, an essential approach for improving their transfection efficiency as gene carriers involves modifying the structure of PAMAM by conjugating functional groups around their surface. In this study, we successfully conjugated an RRHRH oligopeptide to the surface of PAMAM generation 2 (PAMAM G2) to create RRHRH-PAMAM G2. This construction aims to condense plasmid DNA (pDNA) and facilitate its penetration into cell membranes, leading to its promising potential for gene therapy. RRHRH-PAMAM G2/pDNA complexes were smaller than 100 nm and positively charged. Nano-polyplexes can enter the cell and show a high transfection efficiency after 24 h of transfection. The RRHRH-PAMAM G2 was non-toxic to HeLa, NIH3T3, A549, and MDA-MB-231 cell lines. These results strongly suggest that RRHRH-PAMAM G2 holds promise as a gene carrier for gene therapy owing to its biocompatibility and ability to deliver genes to the cell.

## 1. Introduction

Gene delivery is an important area of research and technology that has attracted significant attention in many fields, such as biomedicine, biotechnology, and genetic engineering [1,2,3]. Its importance is reflected in its diverse applications, including diabetes management, vaccine development, infectious disease control, and cancer treatment [4,5,6]. Gene carriers play a crucial role in gene therapy by acting as intermediaries to transport genetic material, such as DNA or RNA, into target-specific locations, whether they are cells or organisms [7,8,9,10,11,12]. Previous studies have indicated that nuclear- or mitochondria-targeting materials can effectively deliver therapeutic genes [13,14,15,16,17]. Efficient gene carriers should not only facilitate gene delivery but also be composed of safe materials [6]. The use of viral vectors for gene delivery has limitations, such as a high product cost, toxicity, and elicitation of host immune responses [18]. Non-viral vectors have received considerable attention as gene carriers because of their ability to overcome these limitations. Nonetheless, the efficacy of non-viral vectors warrants further improvement [19,20].

Cationic polymers, such as chitosan derivatives, polyethyleneimine (PEI), and pol-yamidoamine (PAMAM), play an important role in gene delivery [21,22,23,24,25,26,27]. PAMAM dendrimers have attracted considerable interest because of their unique properties, resulting in their varied applications in gene therapy [26,27]. PAMAM dendrimers have simple but highly branched structures with sufficiently controlled terminal functional groups. Their structure offers the flexibility to modify a specific functional group that can be useful for gene delivery [23]. This may include incorporating a targeting moiety, achieving controlled release, and improving gene loading. Various generations of PAMAM have been explored for their use as gene-delivery vehicles. PAMAM G5 was modified via conjugation with cholesterol, PEG, and folic acid to enhance its efficiency as a gene-delivery system [24]. In addition, numerous specific peptides have been grafted onto the surface of PAMAM G4, resulting in excellent transfection capabilities [25,28,29,30,31]. More recently, less toxic gene-delivery vehicles have been developed using lower generations of PAMAM dendrimers, such as PAMAM G3 and PAMAM G2 [32,33].

The biocompatibility of PAMAM derivatives depends on the type of modification. Short peptides can be conjugated with PAMAM to create gene carriers. Polymer–peptide conjugates can provide novel gene carriers due to their biocompatibility, targeted delivery, stability, and versatility [34,35,36,37,38,39]. Their use in research and therapeutic contexts underscores their significance in advancing gene therapy and improving treatments for various medical conditions. Histidine residues were added to the peptides conjugated to the dendrimer to reduce the toxicity of the polymer [26,33]. Moreover, the introduction of a cathepsin B-sensitive peptide into PAMAM G2 resulted in remarkable transfection efficiency, facilitating controlled release within cancer cells [25]. In addition, nucleus-targeting peptides enhance the effectiveness of gene delivery [30,33]. Therefore, the combination of peptide-modified PAMAM dendrimers leveraged the strengths of both PAMAM dendrimers and peptides to form highly effective and targeted gene delivery systems. These modified carriers offer enhanced biocompatibility, cell penetration, cargo protection, and the ability to deliver genes precisely to specific cell types. This makes them essential components for advancing gene therapy and related research endeavors [26,29,33].

Previous studies have indicated that peptides containing histidine and arginine are well-suited for gene delivery. Histidine exhibits a remarkable ability to induce a “proton sponge effect”, enabling this complex to escape from the endosomes [26,31]. The latest report revealed that histidine residues act as scavengers of the reactive oxygen species (ROS) generated during the transfection process [33]. This scavenging ability helps prevent excessive damage to the mitochondrial membranes, ultimately resulting in reduced cytotoxicity [33]. Arginine, along with its guanidine group, facilitates binding to therapeutic genes and cell membranes. Arginine-rich peptide sequences can efficiently cross the cell membrane [36,40,41,42]. Numerous proteins contain histidine and arginine residues [42,43]. One such protein is the sodium-coupled bicarbonate transporter (NCBE), which contains the peptide sequence RRHRH. NCBE is a member of the SLC4 family of bicarbonate transporters, which play a crucial role in maintaining cellular pH levels. Interestingly, the NCBE exhibited higher expression levels in retinal and brain tissues. 

**Figure 1:**
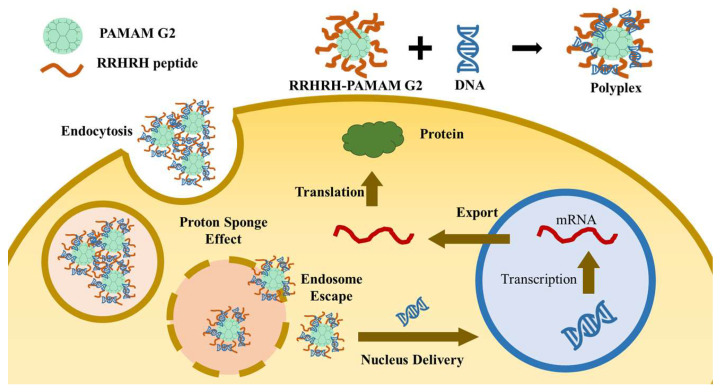
Schematic representation for the preparation of the RRHRH-PAMAM G2/pDNA complex and endocytosis of the polyplexes.

In the present study, the RRHRH oligopeptide was selected as the key component of a biocompatible functional peptide, and the peptide–PAMAM conjugate (RRHRH-PAMAM G2), which was shown to be an efficient gene delivery vehicle (Figure 1). 

## 2. Results and Discussion

### 2.1. The Synthesis of RRHRH-PAMAM G2

**Figure 2:**
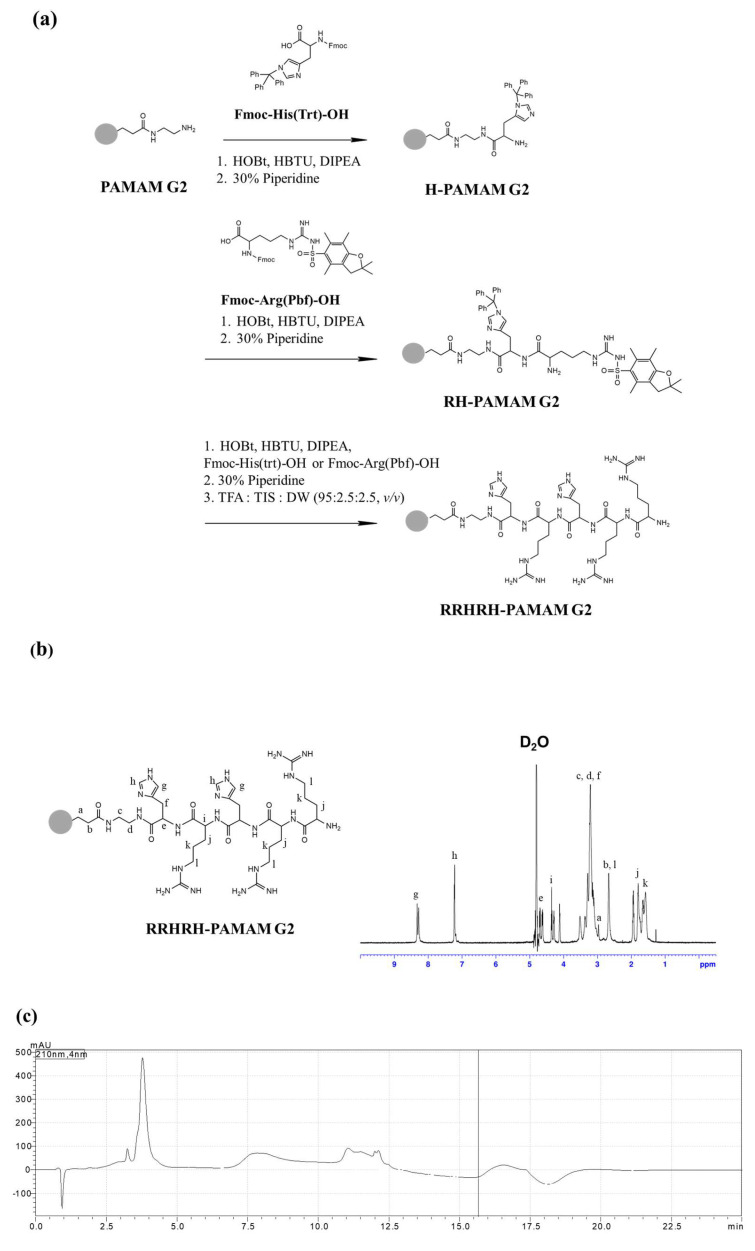

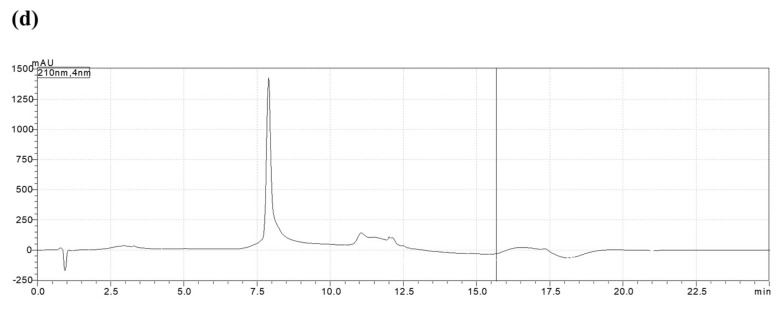
(**a**) A schematic representation of the synthesis of RRHRH-PAMAM G2. (**b**) ^1^H NMR spectra of RRHRH-PAMAM G2. (**c**,**d**) HPLC chromatogram of PAMAM G2 and RRHRH-PAMAM G2.

The histidine–arginine dipeptide effectively improves the delivery efficiency of pDNA by enhancing cellular uptake and capitalizing on the proton sponge effect [26,32]. Consequently, we hypothesized that longer His–Arg dipeptide sequences can exhibit an even higher pDNA delivery efficiency. In this study, we selected the NCBE peptide sequence (RRHRH) containing histidine and arginine to attach to the surface of second-generation polyamidoamine dendrimers (PAMAM G2). The process of attaching histidine and arginine to PAMAM G2 uses HOBT and HBTU as coupling agents (Figure 2a). The resulting RRHRH-PAMAM G2 compound was successfully verified using ^1^H NMR spectroscopy, with a remarkable conjugation yield of 92%. The ^1^H NMR spectra are shown in Figure 2b. The chemical shift (δ) values in ^1^H NMR spectroscopy included 1.63(-CHCH_2_C**H_2_**CH_2_NH- of the arginine unit), 1.79(-CHC**H_2_**CH_2_CH_2_NH- of the arginine unit), 2.66(-NCH_2_C**H_2_**CO- of the PAMAM G2 unit), 2.96(-NC**H_2_**CH_2_CO- of the PAMAM G2 unit), 3.14(-CC**H**_2_C- of the histidine unit), 3.20(-NHCH_2_C**H_2_**NH- of the PAMAM G2 2 unit), 3.36(-COC**H**NH_2_ of the arginine unit), 3.51(-NHC**H_2_**CH_2_NH- of the PAMAM G2 unit), 4.34(-COC**H**NH- of the PAMAM G2 unit), 4.70(-COC**H**NH- of the PAMAM G2 unit), 7.22(-NC**H**NH- of the histidine unit), and 8.32(-NHC**H**C- of the histidine unit). As shown in Figure 2c,d, the product was observed at around 4 and 8 min for PAMAM G2 and RRRHRH-PAMAM G2, respectively, which shows the purity of the dendrimers. The results demonstrate the successful synthesis of RRHRH-PAMAM G2.

### 2.2. The Titration of RRHRH-PAMAM G2

**Figure 3:**
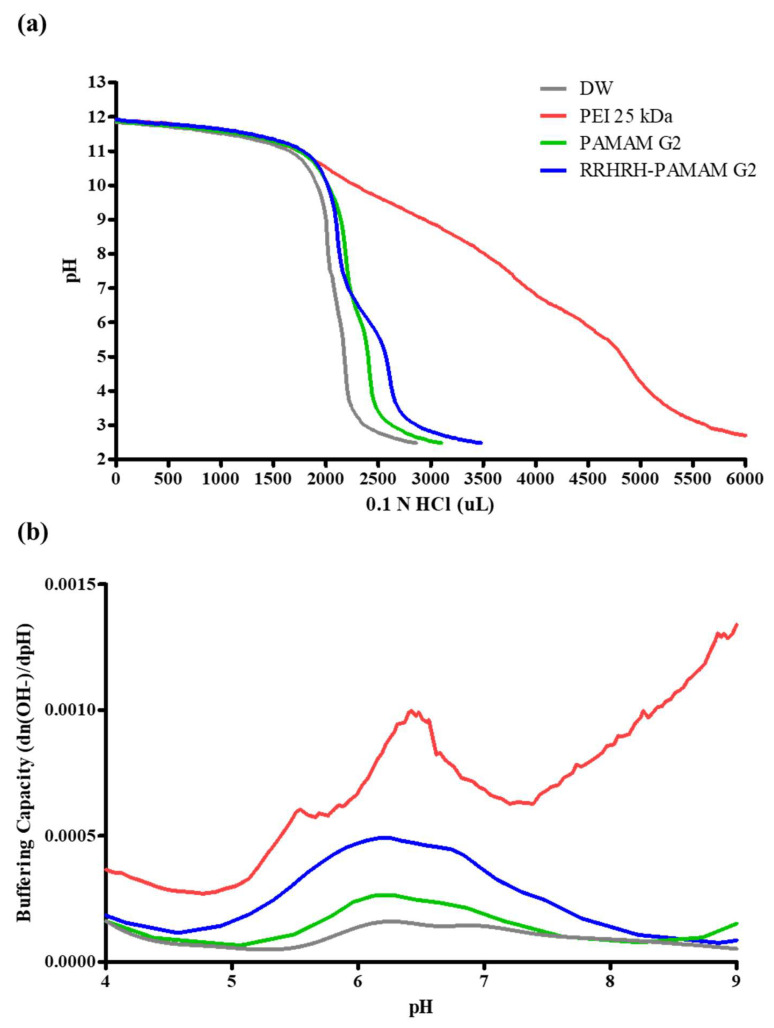
(**a**) The acid–base titration of PEI 25 kDa, PAMAM G2, and RRHRH-PAMAM G2 and (**b**) the buffering capacity of the polymers.

One hypothesis in relation to the endosomal escape of polyplexes is the proton sponge effect, which suggests that chloride ions and water influx occur due to polymer protonation [44]. The RRHRH-PAMAM G2 dendrimer contains multiple histidine residues, and the imidazole groups with a pKa value of six are protonated in an acidic environment [25,31,33]. Consequently, we hypothesized that RRHRH-PAMAM G2 would exhibit the proton sponge effect in the pH ranges that are typically observed for endosomes. The protonation of RRHRH-PAMAM G2 was evaluated by calculating its buffering capacity using the titration method. As shown in Figure 3, PEI 25 kDa exhibited the most robust buffering capacity effect, attributed to the protonation of its imines and amine groups, which possess pKa values in the range of approximately 8.5–9.0; hence, PEI 25 kDa served as a positive control. Water was used as a negative control because of its limited buffering capacity. Both PAMAM G2 and RRHRH-PAMAM G2 dendrimers exhibited proton-buffering effects. However, RRHRH-PAMAM G2 displayed an enhanced buffering capacity within the pH range of 5–7 compared to native PAMAM G2. This suggests that RRHRH-PAMAM G2 polyplexes may effectively overcome the endosomal acidic conditions, potentially leading to improved gene transfection efficiency.

### 2.3. The Complex Test of RRHRH-PAMAM G2

**Figure 4:**
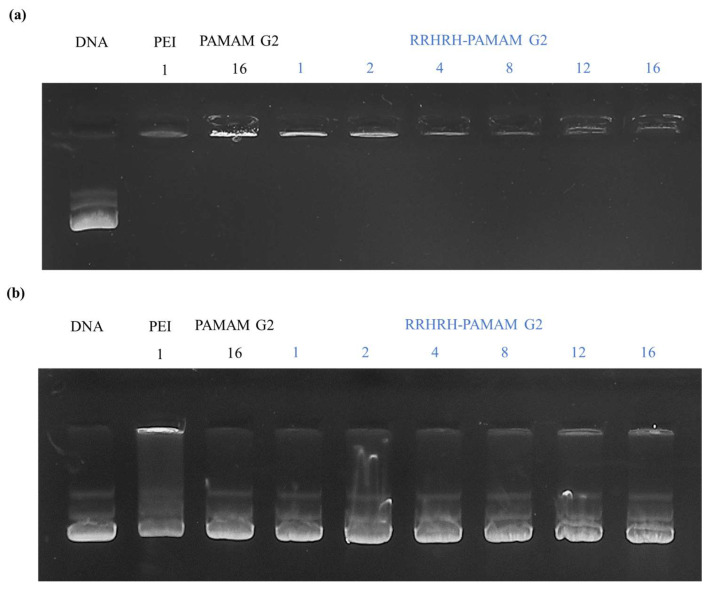
Gel electrophoresis results. (**a**) Agarose gel retardation assay showing pDNA (lane 1), PEI 25 kDa (lane 2), PAMAM G2 (lane 3), and RRHRH-PAMAM G2 polyplexes (lanes 4–9). (**b**) Heparin competitive assay displaying pDNA (lane 1), PEI 25 kDa (lane 2), PAMAM G2 (lane 3), and RRHRH-PAMAM G2 polyplexes (lanes 4–9). In total, 20 µg of heparin was added to each well. Each lane corresponds to a weight ratio of the polymer:pDNA.

The guanidine group in the arginine residue played a vital role in membrane penetration, DNA condensation, and the formation of small polyplexes via electrostatic interactions [26,32,45]. Therefore, RRHRH-PAMAM G2 was considered suitable for comparison with DNA. We performed a gel retardation assay to assess the DNA binding of RRHRH-PAMAM G2. Various polymer-to-pDNA weight ratios were used to prepare the RRHRH-PAMAM G2/pDNA polyplexes, and pDNA migration was examined using agarose gel electrophoresis. Remarkably, the pDNA band disappeared completely at a polymer-to-pDNA weight ratio of 1:1 (Figure 4a). These data confirmed the successful complexation of RRHRH-PAMAM G2 with pDNA at a weight ratio of 1:1 (Figure 4a). Polyplexes were prepared at various polymer/DNA ratios and subsequently incubated with heparin. Heparin was used for competitive assays. The data indicate that DNA was released from the polyplexes, implying that DNA could be liberated within the cell owing to competition with the initial protein. Notably, the polyplexes were not completely released at high polymer/DNA ratios (*w*/*w*) (Figure 4b).

### 2.4. The Characterization of Complexes

**Figure 5:**
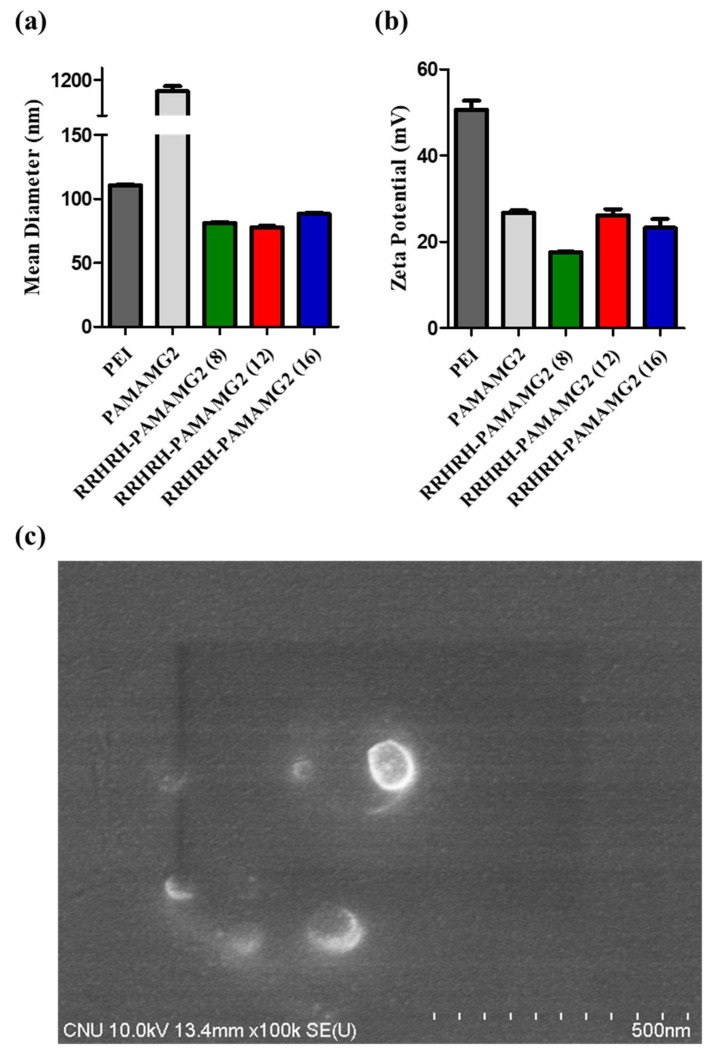
Characterization of polyplexes. (**a**) Average diameter. (**b**) Zeta potential values of PEI 25 kDa/pDNA polyplexes (1:1), PAMAM G2/pDNA polyplexes (16:1), and RRHRH-PAMAM G2/pDNA polyplexes. Data are expressed as mean ± standard deviation (n = 3). (**c**) Morphology of RRHRH-PAMAM G2/pDNA polyplexes was performed using FE-SEM.

For successful gene transfection into cells, the polyplexes must effectively overcome several challenges, including resistance to nucleases and protection against degradation within the endosomal/lysosomal pathways during cellular trafficking [44]. Consequently, the formation of stable and compact polyplexes plays a crucial role in the controlled release and safeguarding of pDNA [25]. The development of cationic polyplexes enables the removal of cell membrane anions, facilitating the reliable release of pDNA into the cytosol. The size and surface charge of RRHRH-PAMAM G2 were determined using dynamic light scattering (DLS) and zeta potential measurements. The PAMAM G2 polyplexes (at a weight ratio of 16:1, *w*/*w*) exhibited an average diameter of 1104.33 ± 59.33 nm, whereas the RRHRH-PAMAM G2/pDNA polyplexes (at a weight ratio of 8:1 to 16:1, *w*/*w*) had diameters <90 nm (Table 1). The RRHRH-PAMAM G2 polyplexes exhibited a low polydispersity index, indicating uniformity and homogeneity. These findings emphasize the ability of RRHRH-PAMAM G2 to form nano-polyplexes. The compact size of these polyplexes facilitated their efficient cellular uptake. PEI 25 kDa/pDNA exhibited a positively charged zeta potential of 53.45 ± 1.35 mV, whereas the polyplexes formed with the PAMAM G2 and RRHRH-PAMAM G2 dendrimers had positively charged zeta potentials of <30 mV (Figure 5 and Table 1). These results indicate the presence of a substantial cationic charge on the polyplexes, which is crucial for cellular internalization. We utilized field emission-scanning electron microscopy (FE-SEM) to investigate the morphology of the polyplexes formed via RRHRH-PAMAM G2. As shown in Figure 5c, the nanoparticles exhibited a spherical shape and had dimensions below 100 nm. 

### 2.5. Intracellular Uptake 

**Figure 6:**
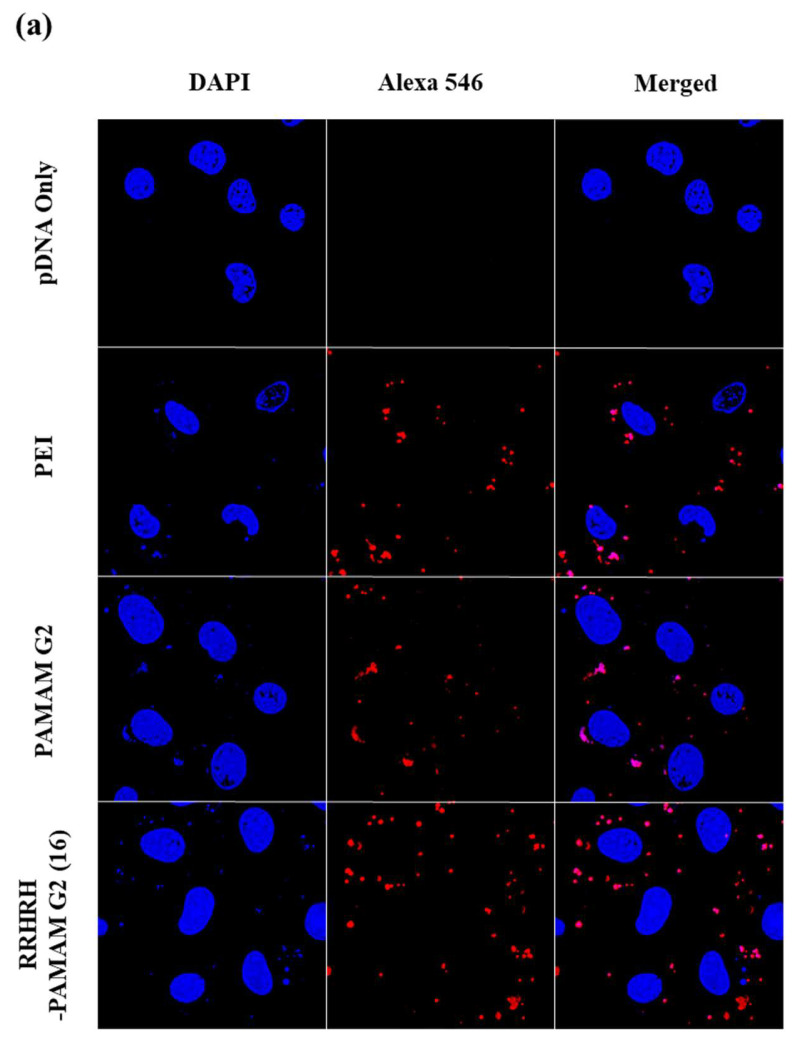

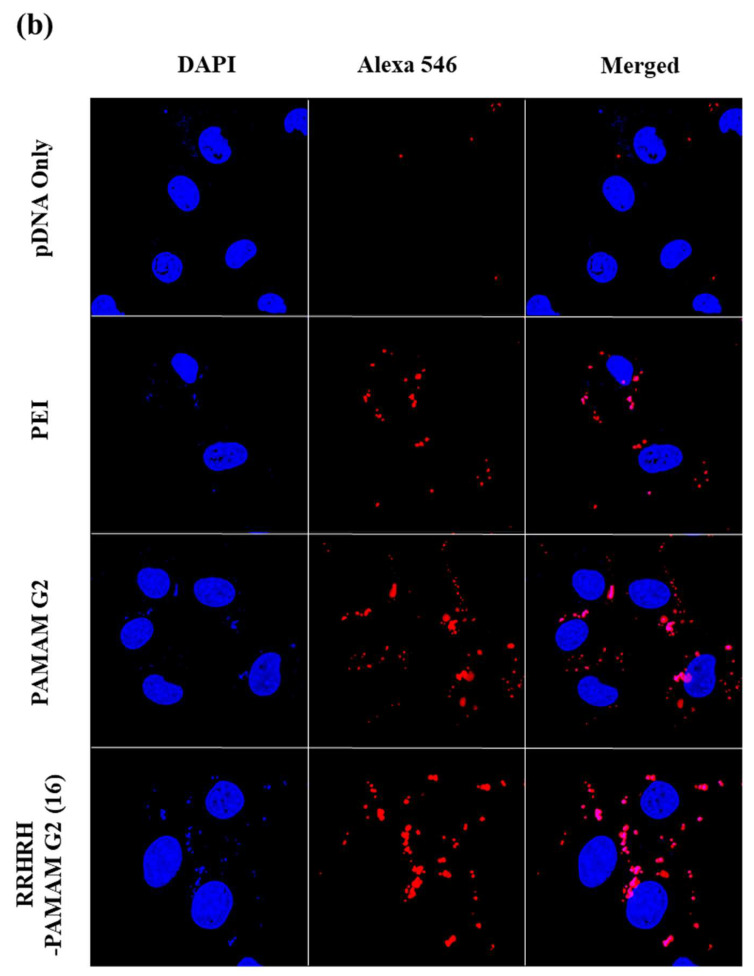
Confocal microscope images (NIH3T3 cells). Polymer concentrations of polyplexes prepared as 2:1 (PEI 25 kDa), 16:1 (PAMAM G2 and RRHRH-PAMAM G2) wt ratios for 12 h (**a**) and 24 h (**b**). Cell nuclei and plasmid DNA were stained with DAPI (blue) and Alexa 546 (red), respectively.

In our investigation of the intracellular uptake of RRHRH-PAM G2 polyplexes, we employed confocal microscopy imaging analysis via labeling plasmid DNA (pDNA) with Alexa Fluor 546, enabling the precise visualization of polyplexes inside a cell. Subsequently, these labeled polyplexes were incubated with NIH3T3 cells over 12 and 24 h to capture detailed images using confocal microscopy. Remarkably, our observations revealed a distinct pattern in the fluorescence signals. While red fluorescence was detected for the polyplexes formed with PEI 25 kDa, PAM G2, and RRHRH-PAM G2 (as shown in Figure 6 and Figure S1), it was the RRHRH-PAM G2 polyplexes that exhibited a significantly stronger and more pronounced red signal, compared to those formed with PAM G2 alone. This difference was evident after 12 h of incubation and became even more prominent after 24 h. The intensity of the red fluorescence indicates a substantial enhancement in cellular uptake specifically facilitated by RRHRH-PAM G2. These results suggest that RRHRH-PAM G2, through its unique composition and design, significantly enhances the cellular uptake of polyplexes, and the efficiency of RRHRH-PAM G2 in promoting cellular internalization can lead to an improved transfection efficiency afterward. This enhanced the cellular internalization capability and holds immense potential for applying RRHRH-PAM G2 as a gene carrier system in gene delivery.

### 2.6. The Transfection

**Figure 7:**
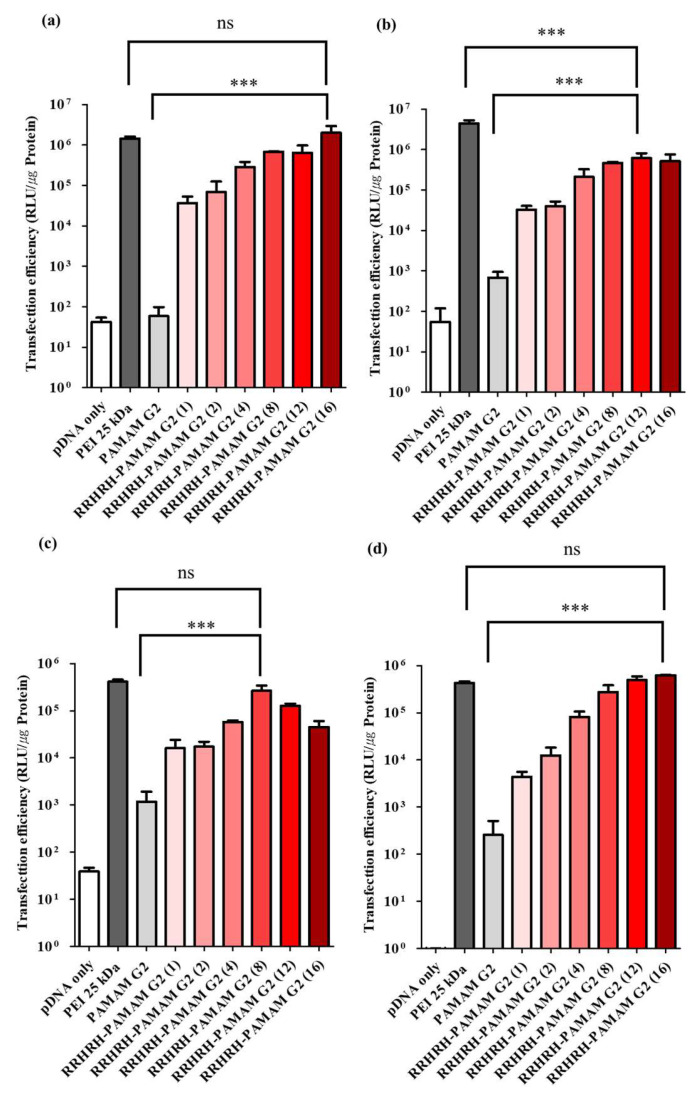
The transfection efficiency of PEI 25 kDa, PAMAM G2 and RRHRH-PAMAM G2 on A549 cells (**a**), HeLa cells (**b**), MDA-MB-231 cells (**c**), and NIH3T3 cells (**d**) for 24 h. Values are expressed as the mean ± SD of independent experiments (n = 3). ns, *p* > 0.05; ***, *p* ≤ 0.001.

To assess the transfection efficiency, we employed the firefly luciferase gene (pCN-luc) as a reporter gene and quantified transfected luciferase activity in diverse cell lines, including HeLa, NIH3T3, A549, and MDA-MB-231. The polyplexes of RRHRH-PAMAM G2 were prepared at varying weight ratios, ranging from 1:1 to 16:1, while branched PEI 25 kDa and PAMAM G2 dendrimers were used as the controls. As shown in Figure 7, RRHRH-PAMAM G2 consistently outperformed PAMAM G2 in terms of its transfection efficiency across all four cell lines. These results can be attributed to the enhanced intracellular uptake, nucleus-localizing feature, and unique buffering capacity inherent to RRHRH-PAMAM G2. The endosome buffering capacity played an essential role in promoting the liberation of polyplexes from the endo/lysosomal compartments, thereby elevating gene expression levels. Importantly, RRHRH-PAMAM G2 can facilitate the escape of polyplexes from endosomes, further enhancing its efficacy in gene delivery. Notably, RRHRH-PAMAM G2 demonstrated a transfection efficiency comparable to that of PEI 25 kDa in NIH3T3, A549, and MDA-MB-231 cells, demonstrating its potential as an alternative gene delivery agent. Although slightly lower than PEI 25 kDa in HeLa cells, this shows the cell line-dependent nature of RRHRH-PAMAM and G2-mediated transfection. In summary, the outstanding transfection efficiency exhibited by RRHRH-PAMAM G2 can be attributed to a combination of factors. These factors include the efficient intracellular uptake of polyplexes and the enhanced proton-buffering capacity of the polymer, which collectively contribute to its remarkable performance.

### 2.7. The Cytotoxicity

**Figure 8:**
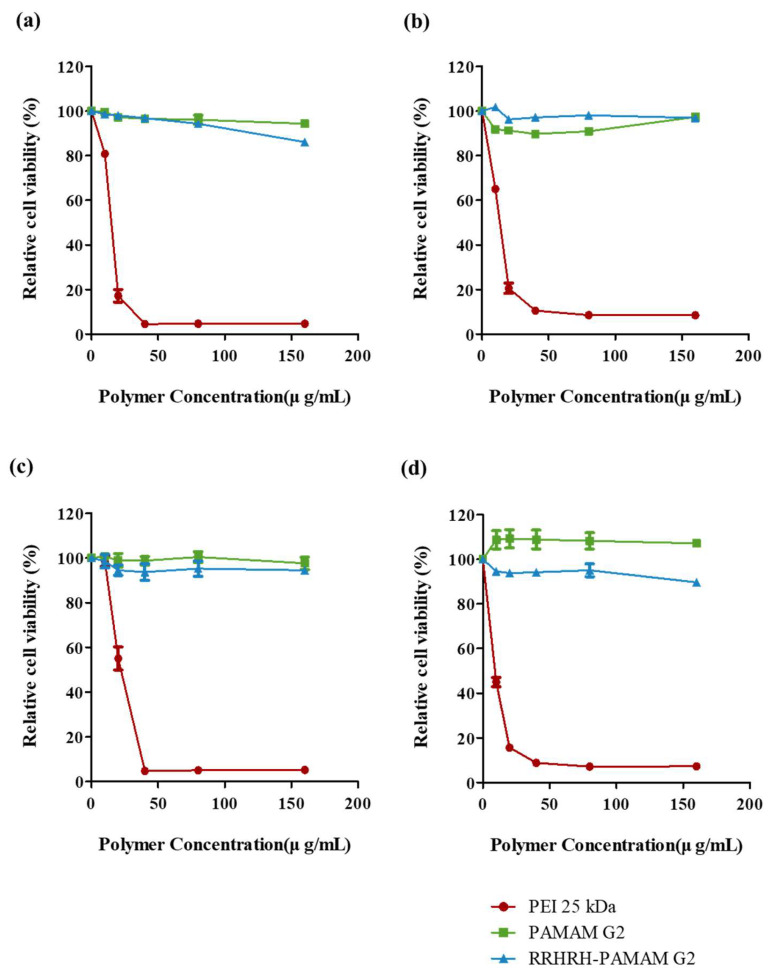
Cell viability of PEI 25 kDa (red circle), PAMAM G2 (green square) and RRHRH-PAMAM G2 (blue triangle) on A549 cells (**a**), HeLa cells (**b**), MDA-MB-231 cells (**c**), and NIH3T3 cells (**d**) for 24 h. Values are expressed as the mean ± SD of independent experiments (n = 4).

The positive charge of polymers or polyplexes substantially contributes to enhanced cell uptake and transfection efficiency. However, an excessive positive charge can lead to cell cytotoxicity due to extensive interactions with cell membrane components, as previously observed with PEI 25kDa [25,26,32]. To assess cytotoxicity, we conducted WST-1 assays at various polymer concentrations using HeLa, NIH3T3, A549, and MDA-MB-231 cells. As shown in Figure 8, PEI 25kDa exhibited pronounced cytotoxicity, whereas both PAMAM G2 and RRHRH-PAMAM G2 demonstrated comparatively low toxicity, even under high-concentration treatments across all cell lines. Notably, previous studies have indicated that peptides containing multiple arginine residues can induce high cellular toxicity owing to their strong interactions with cell membranes. However, our peptide–PAMAM G2 conjugate, comprising three arginine residues, did not exhibit such toxicity. This can be attributed to the presence of histidine residues within the peptide and the use of a low-generation PAMAM dendrimer as the core polymer. Prior studies have indicated that histidine residues can function as scavengers for the generation of reactive oxygen species (ROS) [33]. This scavenging capability helps to mitigate excessive damage to the mitochondrial membranes, ultimately resulting in reduced cytotoxicity. These findings suggest that RRHRH-PAMAM G2 is a biocompatible polymer that is well-suited to therapeutic applications.

## 3. Materials and Methods

### 3.1. Materials

The polyamidoamine dendrimer ethylenediamine core generation 2 solution (PAMAM G2), ninhydrin, N, N-dimethylformamide (DMF), dimethylsulfoxide (DMSO), diisopro-pylethylamine (DIPEA), piperidine, triisopropylsilane (TIS), trifluoroacetic acid (TFA), and Fmoc-Arg(pdf)-OH were purchased from Sigma-Aldrich Korea (Seoul, Republic of Korea). Fmoc-His(trt)-OH, 1-Hydroxybenxotriazole hydrate (HOBt) and 2-(1H benzotria-zole-1-yl)-1,1,3,3-tetramethyluronium (HBTU) were purchased from Novabiochem (Darmstadt, Germany). The luciferase assay system (E1500) was purchased from Promega (Madison, WI, USA). The D-Plus™ CCK cell viability assay kit was purchased from Dongin LS (Hwaseong, Republic of Korea). Bisbenzimide (Hoechst 33342) was purchased from Sigma-Aldrich. The ULYSIS Alexa Fluor 546 nucleic acid labeling kit (U21652) was purchased from Molecular Probes (Eugene, OR, USA). Dulbecco’s phosphate-buffered saline (DPBS) and Dulbecco’s modified Eagle medium (DMEM) were purchased from Welgene (Gyeongsan, Republic of Korea). HeLa, NIH3T3, A549, and MDA-MB-231 were supplied from a Korean cell bank. ^1^H NMR spectra were recorded on a Bruker AVANCE III 600 instrument.

### 3.2. Synthesis of the PAMAM G2 Derivatives

We dissolved 10 mg of PAMAM G2 in a 1:1 (*v*/*v*) mixture of anhydrous DMF and DMSO, and the solution was stirred in the presence of 4 equivalents of HOBt, HBTU, and Fmoc-His(trt)-OH, along with 8 equivalents of DIPEA to primary amines of PAMAM for 16 h at 37 °C. The resulting product was precipitated with cold diethyl ether and centrifuged to remove the supernatant. The precipitate was thoroughly washed with excess diethyl ether and dried under a stream of nitrogen gas. Subsequently, the Fmoc groups of Fmoc-His(trt)-coupled dendrimers were removed by adding 30% piperidine to DMF (*v*/*v*) for 2 h. The mixture was precipitated in cold diethyl ether, washed, and dried under a nitrogen gas stream. Further steps were performed, as described above. Next, to remove the trt- and pbf-protecting groups, the dried compound was dissolved in a TFA/TIS/DW 95:2.5:2.5 (*v*/*v*) mixture, stirred for 6 h at room temperature, and washed with diethyl ether. The precipitate was dissolved in DW and transferred to a dialysis membrane (MWCO 3500, Spectra/Por). After 18 h of dialysis, the product was lyophilized and analyzed using 600 MHz ^1^H NMR spectroscopy and HPLC (Waters, MA, USA). The HPLC followed the previous method [46]. RRHRH-PAMAM G2 was performed at a concentration of 2000 ppm. HPLC analysis was examined using a C18 (5 µm, 2.1 × 100 mm) column with a detector wavelength of 210 nm. The column temperature was maintained at 25 °C, and the flow rate was set to 0.4 mL/min for 10 min. A gradient analysis was conducted, transitioning from a mixture of water with trifluoroacetic acid (H_2_O in 0.1% TFA) to acetonitrile (acetonitrile in 0.1% TFA).

### 3.3. Titration

Acid–base titration was performed to demonstrate the buffering capacity of RRHRH-PAMAM G2 [26,31,33]. We prepared PEI 25 kDa (30 × 10^−8^ M), PAMAM G2, and RRHRH-PAMAM G2 using equivalent amounts. Each sample was then added to 4 mL of a 150 mM NaCl solution containing 100 µL of 1 N NaOH. A control group that used only water was also included. The samples were titrated by adding 20 µL of a 0.1 N HCl solution until the pH value reached 3.0. The pH of the samples was measured using a pH meter (pH 211 microprocessor pH meter; HANA Instruments, Seoul, Republic of Korea). The calculation for the buffering capacity (β) of each polymer was determined using the formula β = dn(OH^−^)/dpH.

### 3.4. Gel Retardation

A gel retardation assay was performed via agarose gel electrophoresis. The polymer and pDNA were combined at weight ratios (polymer pDNA, *w*/*w*) of 1 for PEI 25 kDa, 16 for PAMAM G2, and 1, 2, 4, 8, 12, and 16 for RRHRH-PAMAM G2 to form polyplexes in the HEPES buffer (25 mM, pH 7.4) for 30 min. The prepared samples were loaded onto 0.7% agarose gels containing ethidium bromide. Subsequently, agarose gel electrophoresis was performed at 100 V for 30 min. For the heparin competition assay, the polyplexes were prepared as described above. Heparin (20 µg) was added to each polyplex and incubated for 30 min. The prepared samples were loaded onto 0.7% agarose gel containing ethidium bromide. Subsequently, agarose gel electrophoresis was performed at 100 V for 30 min.

### 3.5. Size and Zeta Potential Measurement

The size and surface charge of the synthesized polyplexes (Section 3.2) were measured using Zetasizer Nano-Zs (Malvent Instruments Ltd., Worcestershire, UK) at room temperature. Measurements were performed in triplicate and expressed as z-averages (sizes).

### 3.6. FE-SEM

The surface morphology of the polyplexes was observed using FE-SEM. The sample was dropped onto a silicon wafer and dried overnight at room temperature. The sample wafer was coated with Pt for 90 s and measured using FE-SEM (S-4800, Hitachi, Japan).

### 3.7. Cell Uptake

The cellular uptake of the complexes was evaluated using confocal microscopy. Labeled pLuc (546-pLuc) was prepared using a ULYSIS^®^ Alexa Flour^®^ 546 nucleic acid labeling kit (U21652), according to the manufacturer’s guidelines. The 546-pLuc/polymer complexes were prepared at the optimal ratios for transfection. The NIH3T3 cells were transfected with 546-pLuc, as described above. After 12 h and 24 h of incubation, the cells were washed with 200 μL DPBS, and the nuclei were stained with DAPI. The 546-positive cells and DAPI signals were observed under a confocal microscope (Carl Zeiss LSM 880 with Airyscan).

### 3.8. Transfection

The HeLa, NIH3T3, A549, and MDA-MB-231 cells were cultured in DMEM containing 10% FBS in an incubator maintained at 37 °C, 5% CO_2_, and 95% humidity. The cells were detached and seeded in 96-well plates with densities of 1.3 × 10^4^ cells/well. The cells were incubated for 24 h before transfection. pLuc/polymer complexes were prepared at various weight ratios and added to the cells (500 ng/well). After 24 h of incubation, the cells were washed twice with DPBS and lysed in a 1× reporter lysis buffer (Promega, Madison, WI, USA). The lysates were centrifuged at 13,200 rpm for 10 min to remove any cell debris. The supernatants were then transferred to new tubes. The protein concentration was measured using a BCA protein assay kit. Luciferase activity was measured using a bicinchoninic acid (BCA) protein assay kit. Luciferase activity was measured using the Luminant LB 9057 luminometer (Berthold Technology, Bad Wildbad, Germany). The final values are presented as the RLU/mg of the total protein.

### 3.9. Cytotoxicity Assay In Vitro

HeLa, NIH3T3, A549, and MDA-MB-231 were used to evaluate the polymer cytotoxicity. The cells were maintained in DMEM containing 10% (*v*/*v*) FBS and 1% (*w*/*v*) penicillin/streptomycin at 37 °C (HeLa, NIH3T3, A549, and MDA-MB-231 cells) in a humidified atmosphere of 5% CO_2_. The cells were seeded at a density of 1.3 × 10^4^ cells/well in a 96-well plate and grown in 100 μL of media for 24 h prior to the incubation with polymers. Thereafter, the cells were treated with 10 µL of the PA-MAM G2 and RRHRH-PAMAM G2, and PEI 25 kDa was used as a positive control. After 24 h of incubation, 10 µL of the EZ-Cytox reagent was added to each well (except for the negative control) and incubated for 2 h. Finally, the absorbance was measured at a wavelength of 450 nm.

### 3.10. Statistical Analysis

The error bars represent standard deviations. A statistical analysis was performed using the GraphPad Prism 5.0. A one-way ANOVA with Tukey Correction was applied to compare multiple conditions. The differences between groups were considered to be significant at a *p*-value of ≤0.05. (ns, *p* > 0.05; ***, *p* ≤ 0.001).

## 4. Conclusions

In summary, this study represents a significant advancement in the field of polymeric gene delivery systems by tailoring poly(amidoamine) (PAMAM) G2 dendrimers through the incorporation of novel peptides that are rich in histidine and arginine residues derived from the NCBE. The features of nano-sized positively charged polyplexes comprising RRHRH-PAMAM G2 and pDNA were confirmed through dynamic light scattering (DLS) and zeta potential measurements, affirming their stability and efficiency. The transfection assays performed using RRHRH-PAMAM G2 for all cells tested demonstrated a remarkable transfection efficiency while maintaining high cell viability. This outcome can be attributed to the strategic incorporation of arginine and histidine residues within RRHRH-PAMAM G2 dendrimers, underlining the importance of this modification to enhance cellular compatibility and reduce cytotoxicity. Furthermore, our findings are substantiated by confocal microscopy, providing compelling visual evidence of the successful internalization of pDNA when complexed with RRHRH-PAMAM G2 dendrimers. This result not only corroborates the efficacy of our engineered dendrimer as a gene carrier but also highlights its potential for targeted gene delivery applications. In conclusion, our study demonstrates the promising potential of the RRHRH-PAMAM G2 dendrimer as a highly efficient and biocompatible carrier for gene delivery.

## Figures and Tables

**Table 1:** The characteristics of polyplexes (polymer/pCN-Luci). Values show the means ± SD (n = 3) of three independent experiments.

Polyplexes (Polymer/pCN-Luci)	Size (nm)	PDI ^a^	Zeta Potential (mV)
PEI	110.87 ± 0.76	0.125 ± 0.006	47.6 ± 1.34
PAMAM G2	1104.33 ± 59.33	0.649 ± 0.082	26.77 ± 0.68
RRHRH-PAMAM G2 (8)	81.22 ± 0.70	0.146 ± 0.006	17.60 ± 0.22
RRHRH-PAMAM G2 (12)	77.90 ± 1.32	0.189 ± 0.011	26.23 ± 1.84
RRHRH-PAMAM G2 (16)	88.40 ± 1.34	0.219 ± 0.015	23.27 ± 2.88

^a^ PDI: polydispersity index.

## Data Availability

Data are contained within the article and Supplementary Materials.

## Supplementary Materials

The following supporting information can be downloaded at: https://www.mdpi.com/article/10.3390/molecules28227644/s1.
