# Supplementary material for: Second-Generation Polyamidoamine Dendrimer Conjugated with Oligopeptides Can Enhance Plasmid DNA Delivery In Vitro
Source: Molecules. 2023 Nov 17;28(22):7644. doi: 10.3390/molecules28227644 (PMC10674462; doi:10.3390/molecules28227644)
Supplement: Supplementary file 1 [file molecules-28-07644-s001.zip › molecules-2696551-supplementary.pdf]

molecules

MDPI

Article

Second-Generation Polyamidoamine Dendrimer Conjugated with Oligopeptides Can Enhance Plasmid DNA Delivery In Vitro

Seongyeon Kim †, Le Thi Thuy †, Jeil Lee and Joon Sig Choi *

Department of Biochemistry, Chungnam National University, 99 Daehak-ro, Yuseong-gu, Daejeon 34134, Republic of Korea; sykim96500@gmail.com (S.K.); ltthuy1990@gmail.com (L.T.T.); jeillee@hongik.ac.kr (J.L.)
* Correspondence: joonsig@cnu.ac.kr; Tel.: +82-42-821-7528; Fax: +82-42-822-7548
† These authors contributed equally to this work.

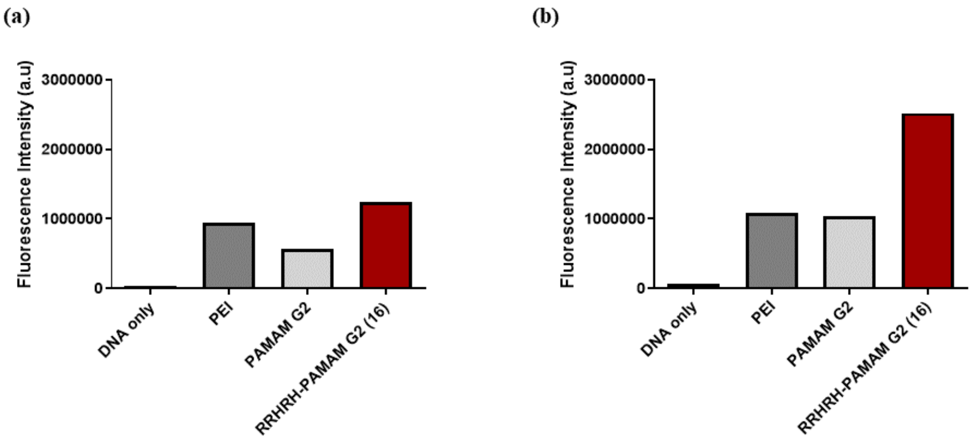

Figure S1. Fluorescence intensity of Alexa 546 with polyplex treatment for 12 h (a) and 24 h (b) in NIH3T3 cells

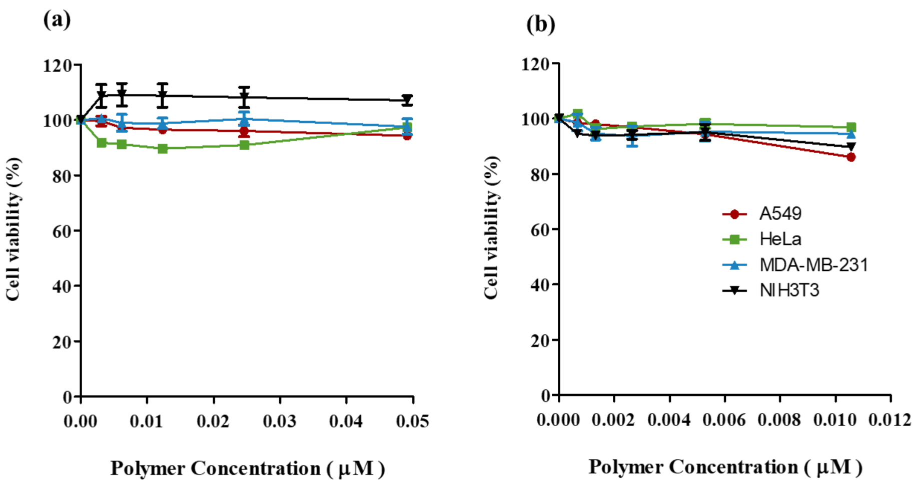

Figure S2. Cell viability of PAMAM G2 (a) and RRHRH-PAMAM G2 (b) in A549, HeLa, MDA-MB-231 and NIH3T3 cells for 24 h.
